# A Multilocus Species Delimitation Reveals a Striking Number of Species of Coralline Algae Forming Maerl in the OSPAR Maritime Area

**DOI:** 10.1371/journal.pone.0104073

**Published:** 2014-08-11

**Authors:** Cristina Pardo, Lua Lopez, Viviana Peña, Jazmin Hernández-Kantún, Line Le Gall, Ignacio Bárbara, Rodolfo Barreiro

**Affiliations:** 1 Departamento de Biología Animal, Biología Vegetal y Ecología, Facultad de Ciencias, Universidade da Coruña, A Coruña, Spain; 2 Phycology Research Group, Ghent University, Ghent, Belgium; 3 Unité Mixte de Recherche 7205, Equipe “Exploration, Espèces et Evolution”, Muséum National d’Histoire Naturelle, Paris, France; 4 Irish Seaweed Research Group, National University of Ireland, Galway, Ireland; Consiglio Nazionale delle Ricerche (CNR), Italy

## Abstract

Maerl beds are sensitive biogenic habitats built by an accumulation of loose-lying, non-geniculate coralline algae. While these habitats are considered hot-spots of marine biodiversity, the number and distribution of maerl-forming species is uncertain because homoplasy and plasticity of morphological characters are common. As a result, species discrimination based on morphological features is notoriously challenging, making these coralline algae the ideal candidates for a DNA barcoding study. Here, mitochondrial (COI-5P DNA barcode fragment) and plastidial (*psb*A gene) sequence data were used in a two-step approach to delimit species in 224 collections of maerl sampled from Svalbard (78°96’N) to the Canary Islands (28°64’N) that represented 10 morphospecies from four genera and two families. First, the COI-5P dataset was analyzed with two methods based on distinct criteria (ABGD and GMYC) to delineate 16 primary species hypotheses (PSHs) arranged into four major lineages. Second, chloroplast (*psb*A) sequence data served to consolidate these PSHs into 13 secondary species hypotheses (SSHs) that showed biologically plausible ranges. Using several lines of evidence (e.g. morphological characters, known species distributions, sequences from type and topotype material), six SSHs were assigned to available species names that included the geographically widespread *Phymatolithon calcareum, Lithothamnion corallioides,* and *L. glaciale*; possible identities of other SSHs are discussed. Concordance between SSHs and morphospecies was minimal, highlighting the convenience of DNA barcoding for an accurate identification of maerl specimens. Our survey indicated that a majority of maerl forming species have small distribution ranges and revealed a gradual replacement of species with latitude.

## Introduction

Maerl or rhodolith beds are accumulations of slow-growing, unattached non-geniculate (non-articulated) coralline algae that build three-dimensional habitats [1] that accommodate a wide biodiversity and are, therefore, considered as hotspots of marine life [2]. Commercial dredging together with a range of indirect impacts (bottom-fishing, aquaculture, eutrophication, sediment dredging) are known to negatively affect their conservation and structure [3]. As a result, maerl beds are listed as threatened and/or declining habitats by OSPAR (The Convention for the Protection of the marine Environment of the North-East Atlantic) [4] and treated as Special Areas of Conservation by EU Habitats Directive (Annex I, categories “sandbank covered by seawater all the time” and “large shallow inlets and bays”). In addition, the two coralline algal species commonly regarded as the main constituents of maerl beds in Europe (*Phymatolithon calcareum* (Pallas) W.H. Adey & D.L. McKibbin and *Lithothamnion corallioides* (P.L. & H.M. Crouan) P.L. & H.M. Crouan) are listed in Annex V as species whose eventual exploitation must be compatible with maintaining a favorable conservation status.

Maerl beds are widely distributed along the coasts of the North-East Atlantic protected by the OSPAR Convention (OSPAR maritime area) and the adjacent Macaronesia. They are particularly frequent in Scotland, Ireland, Brittany and Galicia [4] at depths ranging from the intertidal to 50 m, but they reach up to 60 min the Canary Islands and Madeira [4]–[7]. Up to 24 species of maerl have been recorded along the OSPAR area and southern adjacent regions (Madeira Archipelago and Canary Islands) (Table 1). However, the actual number might be smaller as the taxonomic validity of eight taxa seems dubious because they were only reported in pioneer works from the 19^th^ century and early 20^th^ (*Lithothamnion breviaxe* Foslie, *L. fornicatum* Foslie, *L. fruticulosum* (Kützing) Foslie, *L. intermedium* Kjellman, *L. nodulosum* Foslie, *L. norvegicum* (Areschoug) Kjellman, *L. tusterense* Foslie and *L. ungeri* Kjellman). Maerl-forming algae belong to six genera (*Lithothamnion, Lithophyllum*, *Mesophyllum, Neogoniolithon, Phymatholithon* and *Spongites*) from two families (Corallinaceae and Hapalidiaceae) within the order Corallinales (Rhodophyta). According to the literature, four species are widely distributed and seemingly follow a latitudinal replacement cline: *Lithothamnion tophiforme* (Esper) Unger and *Lithothamnion glaciale* Kjellman are mostly arctic and subarctic species, while *P. calcareum* and *L. corallioides* occur from the North and Celtic Seas to Madeira-Canary Islands (*L. corallioides*) or Azores (*P. calcareum*). The remaining 12 species occupy narrower latitudinal ranges. Eight of them were reported for regions with a long tradition of taxonomic surveys: Scotland, Britain, Ireland, and French Brittany (*Lithothamnion lemoineae* Adey, *L. sonderi* Hauck, *Phymatolithon purpureum* (P.L. Crouan & H.M. Crouan) Woelkerling & L.M. Irvine, *Mesophyllum lichenoides* (J. Ellis) Me. Lemoine, *Lithophyllum dentatum* (Kützing) Foslie, *L. duckerae* Woelkerling, *L. fasciculatum* (Lamarck) Foslie and *L. hibernicum* Foslie). The remaining four include species reported for Macaronesia (*Neogoniolithon brassica-florida* (Harvey) Setchell & L.R. Mason, *Lithophyllum crouanii* Foslie, *Spongites fruticulosa* Kützing) plus *Mesophyllum sphaericum* V. Peña, Bárbara, W.H. Adey, Riosmena-Rodríguez & H.G. Choi, a maerl alga known from a single location in Galicia. An overwhelming majority of the previous studies have entirely relied on traditional practices of taxonomy based on morphological/anatomical characters even though morphological identification of non-articulated coralline algae is challenging because phenotypic plasticity and convergence have resulted in a lack of well-defined diagnostic characters [8]. Only very recently, DNA information has been used to identify and delineate European maerl-forming species [9] shedding light on our fragmentary knowledge on alpha diversity and genuine distribution of maerl-forming species.

**Table 1:** Distribution of maerl-forming species reported in the literature for OSPAR regions and southern adjacent areas.

	OSPAR Region												Adjacent areas	
	I			I–II	II–III		III	II–IV	IV			V		
	Svalbard Archipelago	Greenland	Iceland	Scandinavia	Scotland	Britain	Ireland	Brittany	Bay of Biscay	Galicia	Portugal	Azores	Madeira	Canary Islands
Widespread maerl-forming species														
*Lithothamnion tophiforme*	[4]	[4], [47], [64]	[65]–[67]	[4], [66], [68]	-	-	-	-	-	-	-	-	-	-
*Lithothamnion glaciale*	[36], [69]	[4], [70], [71]	[66], [67], [70]	[4], [66], [68], [70]	[4], [44], [70], [72]	[4], [44], [72]	[44], [72]	-	-	-	-	-	-	-
*Phymatolithon calcareum*	-	-	-	[4], [66], [73]	[74]	[35], [44], [72]	[44], [72]	[4], [75], [76]	[77]	[1], [78], [79]	[7]	[80]	-	-
*Lithothamnion corallioides*	-	-	-	[68]	-	[4], [44], [68]	[4], [44], [72]	[4], [75], [81]	-	[1], [78]	-	-	[6]	[4], [5]
Minor maerl-forming species (narrow or uncertain distribution)														
*Lithothamnion fruticulosum*	-	[68]	[65]	[68]	-	[68]	[82]	-	-	-	-	-	-	-
*Lithothamnion breviaxe*	-	-	-	[68]	-	-	-	-	-	-	-	-	-	-
*Lithothamnion fornicatum*	-	-	-	[68]	-	-	-	-	-	-	-	-	-	-
*Lithothamnion intermedium*	-	-	-	[69], [83]	-	-	-	-	-	-	-	-	-	-
*Lithothamnion nodulosum*	-	-	-	[68]	-	-	-	-	-	-	-	-	-	-
*Lithothamnion tusterense*	-	-	-	[83]	-	-	-	-	-	-	-	-	-	-
*Lithothamnion ungeri*	-	-	-	[68], [69]	-	-	-	-	-	-	-	-	-	-
*Lithothamnion norvegicum*	-	-	-	[69], [83]	[83]	-	-	-	-	-	-	-	-	-
*Lithothamnion lemoineae*	-	-	-	-	[4], [44]	-	-	-	-	-	-	-	-	-
*Lithothamnion sonderi*	-	-	-	-	[4]	-	-	-	-	-	-	-	-	-
*Phymatolithon purpureum*	-	-	-	[72]	-	-	[44], [72]	-	-	-	-	-	-	-
*Lithophyllum duckerae*	-	-	-	-	-	[44]	-	-	-	-	-	-	-	-
*Lithophyllum hibernicum*	-	-	-	-	-	-	[4], [44], [84]	-	-	-	-	-	-	-
*Mesophyllum lichenoides*	-	-	-	-	-	-	[45]	-	-	-	-	-	-	-
*Lithophyllum fasciculatum*	-	-	-	-	-	[4], [44]	[4], [44], [85]	[4], [81]	-	-	-	-	-	-
*Lithophyllum dentatum*	-	-	-	-	-	-	[4], [44], [86]	[4]	-	-	-	-	-	-
*Mesophyllum sphaericum*	-	-	-	-	-	-	-	-	-	[9]	-	-	-	-
*Neogoniolithon brassica-florida*	-	-	-	-	-	-	-	-	-	-	-	[80]	-	-
*Lithophyllum crouanii*	-	-	-	-	-	-	-	-	-	-	-	[80]	-	-
*Spongites fruticulosa*	-	-	-	-	-	-	-	-	-	-	-	-	[6]	-

Records restricted to reports as maerl; some species have wider reported ranges as encrusting forms. Currently accepted names are used except for *Lithothamnion fruticulosum* where an older name is retained (see further details in [87]).

The onset of the 21^st^ century has witnessed notable technological advances that can facilitate and accelerate the description of biodiversity [10], [11]. In particular, DNA barcoding (http://www.ibol.org/) employs short, standardized DNA fragments as a diagnostic tool for identifying species [12]. In Rhodophyta, DNA barcodes obtained by sequencing the 5’ end of the mitochondrial gene cytochrome oxidase I (COI-5P) [13], [14] proved very effective to shortcut the difficulties of morphology-based identification, allowing an accurate identification of known species [15]–[23] and/or the detection of cryptic ones [16], [20], [24]–[26]. In comparison, COI-5P sequences have been less frequently used to delineate new species of red algae [15], [18], [20], [22], [25]. Indeed, when DNA barcoding suggested the existence of new species, it was rarely regarded as a definitive proof; instead, it was used along with other genetic, morphological, geographical or ecological features in what has been referred to as integrative taxonomy [10], [27], [28].

Despite the above, DNA barcodes have been used as an exploratory tool for poorly surveyed taxa provided that the groups delineated by barcodes are regarded as primary species hypothesis (PSHs) [29], [30]. PSHs can then be further tested with other sources of molecular, morphological, geographical and/or ecological evidence and even a multistep approach has been proposed to turn PSHs into more conclusive secondary species hypotheses (SSHs) [10] (for a similar approach see the molecular-assisted alpha taxonomy in [26]). In this context, the initial step is crucial and consists of the partition of COI-5P sequences into a set of PSHs. Recently, two methods based on distinct criteria have been proposed to infer the limits of the various PSHs when only molecular data are available and with no need for prior assumptions. On the one hand, the Automatic Barcode Gap Discovery (ABGD) [30] is a fast method that uses distances to split the sequence alignment into a set of PSHs following a recursive procedure until there is no further partitioning. This procedure automatically finds breaks in the distribution of genetic pairwise distances, referred to as the ‘barcode gap’, even when intra- and interspecific distances overlap. On the other hand, the General Mixed Yule Coalescent (GMYC) model [29] is based on detecting the shift of the branching rate that takes place in clock-constrained calibrated trees at the point of transition from species-level (speciation) to population-level (coalescence) evolutionary processes. Using a likelihood criterion, the GMYC method permits an automated species delineation with appropriate statistical measures of confidence. A later extension of the method allows for a variable transition from coalescent to speciation among lineages [31]. GMYC has been shown to be robust to a range of departures from its assumptions (varying population sizes among species, alternative scenarios for speciation/extinction, population growth and subdivision within species) but the accuracy of its delimitations can be compromised in groups with large effective population sizes and short divergence times between species [32]. Other potential shortcomings of the GMYC method have been extensively discussed elsewhere [33].

In this study, COI-5P sequences were obtained for maerl-forming species along the OSPAR maritime area and the adjacent Macaronesia. DNA barcodes were used to delimit a set of PSHs that were subsequently corroborated or challenged with independent molecular, geographic, and morpho-anatomical evidence.

## Materials and Methods

### Study area and sample collection

As the study did not involve endangered or protected species, no specific permissions were required for sampling at most locations (see Table S1 for coordinates). Still, sampling at two locations situated within a national park in NW Spain (lat 42.211° long −8.896° and lat 42.394° long −8.815°) was conducted with the permission of the park authority (Parque Nacional Marítimo Terrestre de las Islas Atlánticas de Galicia) and the park authority has signed a document stating its interest in the results of this study.

Collection information for all the specimens used in this study is available at the Barcode of Life Data Systems (BOLD: www.boldsystems.org; project “maerl-NE Atlantic”). From 1999 to 2011, maerl specimens were extensively sampled by SCUBA diving or dredging within 4 out of the 5 regions of the OSPAR maritime area (Table S1); sampling ranged from the low intertidal to 40 m depth. Despite our efforts, no sample could be obtained for region V where maerl beds are probably restricted to the Azores Archipelago. To circumvent this shortage, samples were collected from the other two Macaronesian Archipelagos: the Canaries and Madeira. Sampling sites included type/neotype localities for 3 out of the 4 widely distributed maerl-forming species: *L. corallioides* (Rade de Brest, Finistere, France [34]), *P. calcareum* (Falmouth Harbour, Cornwall, England [35]), and *L. glaciale* (Spitsbergen Island, Svalbard Archipelago [36]). Additionally, our samples included holotype material of the recently described *M. sphaericum* from the herbarium SANT of Universidad de Santiago de Compostela [9] and neotype material of *P. calcareum* from the herbarium BM of the British Museum of Natural History [35].

Freshly collected material was transported to the laboratory in seawater, oven-dried or air-dried as soon as possible, and vouchered in silica. Vouchers were temporarily deposited in a personal collection (BioCost Research Group, University of A Coruña, Spain) and will be transferred to the official SANT Herbarium. When feasible, several specimens per morphotype (differences in size, shape, branch thickness, and general habit) were sequenced at each locality. This sampling regime was intended to maximize the detection of species encountered at the various collecting sites while keeping the sequencing effort to a reasonable size. All specimens were photographed and identified to the lowest taxonomic level possible using morphology-based keys and specialized literature. A selection of specimens was also examined under scanning electron microscope (SEM, model JEOL JSM 6400, University of A Coruña).

### Field identification

A putative species name was assigned to all specimens based on their gross morphology. Our 224 collections were partitioned into 10 different morphospecies belonging to 4 genera and 2 families (Hapalidiaceae and Corallinaceae). Most plants were identified either as *P. calcareum* (140 collections) or as *L. corallioides* (60), two main constituents of maerl in Atlantic Europe. A much smaller number of collections fitted the description of *L. glaciale* (9), *M. sphaericum* (4), *L. dentatum* (2), and *L. fasciculatum* (1). Finally, eight plants exhibited external features typical of the genus *Lithothamnion*; however, none of these plants exhibited diagnostic characters necessary for their identification at the species level. Nevertheless, based on some morphological distinctions, they were partitioned into four morphospecies temporarily labeled as *Lithothamnion* sp1 (3 collections), *Lithothamnion* sp2 (1), *Lithothamnion* sp3 (1), and *Lithothamnion* sp4 (3).

### DNA extraction, PCR amplification and sequencing

A subsample for DNA extraction was obtained by grinding a portion of the living surface of each specimen after avoiding areas with epiphytes, animal structures, and/or damaged tissue. Special cautions were taken with the neotype of *P. calcareum* because this specimen has been archived in BM since 1983 [35]. To avoid contamination, this archival specimen was processed (DNA extraction and PCR amplification) individually with fresh batches of reactants on a separate date after carefully cleaning the laboratory. To increase the possibility of detecting contamination, several genes were amplified for this specimen on the same date (SSU, *rbc*L, *psbA*, COI-5P) running negative controls in parallel for each gene; none of the chromatograms showed evidence of background signal and all negative controls were clean [37]. The holotype of *M. sphaericum* in SANT was collected shortly before the present study (October 2008) and processed alongside topotype material of the same species. Attempts to acquire sequence data from type material of other species included *L. corallioides*, *L. fornicatum, N. brassica-florida*, and *S. fruticulosa* but proved unsuccessful.

DNA was extracted with the DNeasy Blood & Tissue Kit Spin-Column Protocol (Qiagen) following manufacture’s recommendations. Two gene fragments were amplified: (i) a fragment of 664 bp of the standard DNA barcode (the 5’ end of the mitochondrial gene cytochrome oxidase I, COI-5P) with primers GazF1 and GazR1 from [14], and GCorR3 (5’TGATTYTTYGGACATCCTGA3’), and (ii) a fragment of 892 bp of the plastidial gene photosystem II reaction center protein D1 (*psb*A) with primers *psb*A-F1 and *psb*A-R2 from [38]. PCR reactants were prepared in a laminar flow hood and PCRs were performed in 25 µL containing 2 µL of DNA template, 2.5 µL of 1× PCR buffer, 2.5 mM MgCl2, 0.192 mM dNTPs, 0.1 µM of each primer, and 1.2 U of Taq DNA Polymerase (Sigma-Aldrich) in a Biometra TProfesional Basic thermocycler following [39]. Amplification success was evaluated by electrophoresis. After removing the excess of primers and nucleotides with shrimp alkaline phosphatase and exonuclease I enzymes, PCR products were bidirectionally sequenced at Macrogen facilities (http://www.macrogen.com). All sequences are publically available in BOLD and GenBank (see Table S1 for BOLD IDs and GenBank accession numbers).

### Data analyses

Sequences were aligned and edited using the program Geneious 5.6.6. As we aimed to delimit species based on sequence data rather than to assess their phylogenetic relationships, we chose not to run maximum-likelihood or maximum parsimony analyses. Instead, COI-5P sequences were partitioned into a set of PSHs using two bioinformatics tools: ABGD [30] and GMYC [29], [31]. For ABGC, genetic distances between specimens were calculated using the Kimura two parameters (K2P) model, a standard metric in DNA barcoding studies. ABGD was remotely run at http://wwwabi.snv.jussieu.fr/public/abgd/abgdweb.html using default values except for the relative gap width (Χ) which was set to 10 to avoid the capture of small local gaps. For the GMYC method, duplicate haplotypes were removed from the alignment using DnaSP 5.10.01 [40]. Since the GMYC method is based on branching rates, branch lengths were estimated under a relaxed log-normal clock with the Bayesian analysis implemented in BEAST 1.7.4. Following [31], BEAST was run using a coalescent (constant population size) prior and the best-fitting model identified by jModelTest (HKY+G with G = 0.153) [41], [42]; the parameters for the substitution model (substitution rate, rate heterogeneity, and base frequencies) were unlinked across positions. MCMC chains were run for 20 million generations with a 10% burnin (determined by visual inspection of MCMC progression). After termination, the MCMC output was analyzed with TreeAnnotator 1.7.4 using all trees after the burnin, a posterior probability limit of 0.5, targeting the maximum clade credibility tree, and keeping the target node heights. Both the single-threshold and the multiple-threshold versions of the GMYC model [29], [31] were optimized onto the output tree with the help of the SPLITS v.1.0-19 package for R. AIC-based support values for the GMYC clusters were calculated following [32]. BEAST and TreeAnnotator were also employed to reconstruct a phylogeny for the *psb*A gene with the same options used for the COI-5P sequences but a different best-fitting model (GTR+G with G = 0.175).

## Results

### Primary Species Hypothesis delineation based on COI-5P sequence data

**Figure 1:**
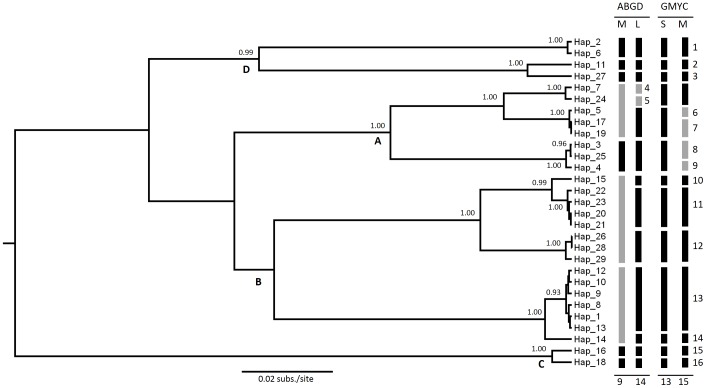
Primary Species Hypothesis (PSHs) delineated with the COI-5P gene. Bayesian gene tree with posterior probabilities (>0.9) next to each node. Branch tips are the 29 haplotypes detected in the study. Vertical thick lines indicate PSHs delineated with ABGD and GMYC methods; numbers next to the vertical lines are PSH codes; letter A–D next to some nodes indicate major lineages. For ABGD, partitions for the more inclusive (M) and less inclusive (L) results are shown. GMYC partitions include the single-threshold (S) and multiple-threshold (M) variants of the method. Grey thick lines indicate discrepancies between partitions.

The 224 collections of maerl were sequenced for a 664 bp fragment of the barcoding COI-5P gene; 29 unique haplotypes were found with 227 variable sites. Genetic pairwise K2P distances ranged from 0 to 0.21 while the shape of the pairwise distance distribution was clearly bimodal with two conspicuous peaks at pairwise distances <0.01 and 0.14–0.16 separated by a rough gap of very low frequencies. The number of PSHs delineated with the ABGD method varied with the maximum prior distance (*P*) used in the analysis. Extreme prior thresholds led to uninformative partitions where either each haplotype was delimited as a different species or all haplotypes were included into a single PSH. Intermediate values of *P* led to partitions with 9 (*P* = 0.013), 13 (*P* = 0.008) and 14 PSHs (*P* = 0.0017 to 0.005). Partitions with 9 and 14 PSHs are detailed in Fig. 1.

GMYC was applied to a phylogenetic tree reconstructed with a relaxed lognormal clock. Effective sample size for each statistic of the tree always was >500 and the MCMC converged to a stationary distribution. The likelihood of the null model (*L_0_* = 170.19) was significantly lower than the maximum likelihood of the single-threshold version of GMYC model (*L*_single_ = 177.97, *P*-value = 0.0004). According to the latter, the transition from speciation to coalescent occurred at a depth of 0.0014 substitutions per site and resulted in a partition with 13 PSHs (confidence interval 4–14): 7 distinct clusters plus 6 singletons (Fig. 1). The likelihood of the multiple-threshold version of the model (*L*_multiple_ = 178.36) also was significantly higher than that of the null model (*P*-value = 0.00028). This version detected a second threshold for the speciation-coalescent transition towards the tips of the tree at an extremely shallow depth of only 0.00026 substitutions per site. With this new threshold, the analysis delimited 15 PSHs (confidence interval 4–15): 7 clusters plus 8 singletons. The mean support value across GMYC clusters was similar in the single-threshold (0.73±0.138) and in the multiple-threshold (0.74±0.237) methods. Nonetheless, the two new clusters delimited by the multiple-threshold algorithm had very little support (<0.45).

Although based on entirely different criteria, the partitions delineated by ABGD and GYMC were notably congruent. The less inclusive partition obtained by ABGD (14 PSHs) was nearly identical to the one produced by the single-threshold version of the GMYC model (13 PSHs). The only discrepancy involved haplotype Hap_24 (a specimen from Svalbard Archipelago) which was resolved as a singleton by ABGD while the GMYC model clustered it with other collections from Svalbard and Scandinavia (Hap_7). In comparison, the more inclusive partition of ABGD (9 PSHs) and the multiple-threshold GMYC (15 PSHs) showed more discrepancies. However, their conflicting PSHs (greyed in Fig. 1) seemed biologically implausible because (i) the more inclusive hypothesis of ABGD clustered groups of haplotypes separated by average K2P distances as large as 0.064 (PSHs 4 to 7 in Fig. 1) or 0.081 (PSHs 10 to 12), and (ii) the conflicting PSHs in the multiple-threshold version of GMYC were separated by distances as small as 0.002–0.005 (sequence divergence between and within PSHs is shown in Table S2a).

### COI-5P and psbA phylogenies and secondary species hypothesis

The phylogeny inferred from the mitochondrial COI-5P gene was well resolved (15 nodes with posterior >0.95 out of 26 nodes) and four major lineages could be distinguished (Fig. 1). Regardless of the approach used for species delineation, all the PSHs that included more than one COI-5P haplotype always coincided with clades with high statistical support (posterior >0.9) with the exception of PSH 7.

**Figure 2:**
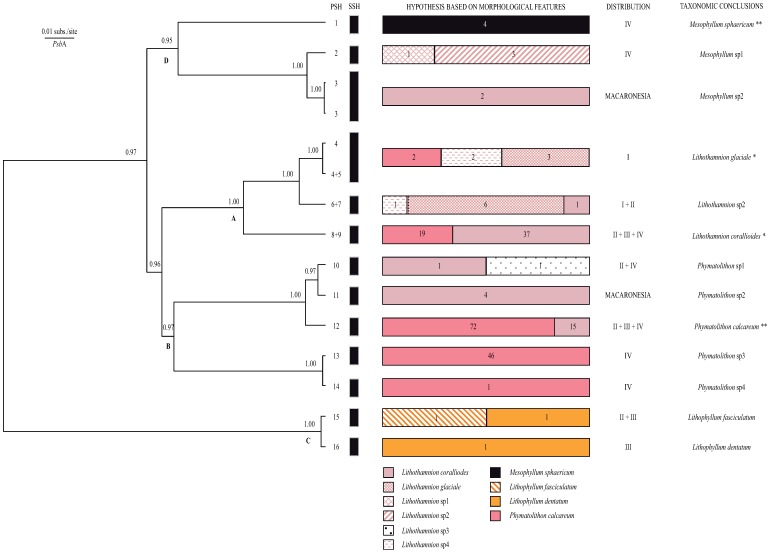
Secondary Species Hypothesis (SSHs) corroborated with the *psb*A gene. Bayesian gene tree with posterior probabilities (>0.9) next to each node. Branch tips are the 16 *psb*A haplotypes detected in the study. Numbers at the tip of the branches are Primary Species Hypothesis (PSH) codes (see Fig. 1) while vertical thick lines delineate SSHs; letters A–D next to some nodes indicate major lineages. Stacked horizontal bars next to the gene tree indicate the morphospecies identified at the onset of the study; numbers within bars are the actual number of specimens recorded for each morphospecies. The distribution of each SSH across OSPAR regions and its taxonomic identity is also provided. * topotype specimens sequenced, ** type specimen sequenced.

The chloroplastic *psb*A gene was sequenced for fifteen of the sixteen PSHs defined with COI-5P data. A fragment of 892 bp generated for 29 specimens produced 15 haplotypes. The phylogeny inferred from *psb*A data was remarkably congruent with the one inferred from the COI-5P gene (Fig. 2). Again, four major lineages could be recognized, one of them separated from the others at an earlier time. Eleven PSHs were characterized by unique *psb*A haplotypes. The remaining four PSHs shared *psb*A haplotypes by pairs (PSH 4–5, PSH 6–7); these pairs corresponded to those cases where the delineation produced by ABGD was in conflict with the solution of the GMYC method. Most *psb*A haplotypes exhibited pairwise distances within a range of 10 (equivalent to 98.8% similarity) to 113 differences (86.6%). Still, a few PSHs were characterized by *psb*A sequences separated by distances as small as 2 (PSH 13 vs. PSH 14, 99.8% similarity), 3 (PSH 15 vs. PSH 16, 99.6%) or 5 point mutations (PSH 10 vs. PSH 11, 99.4%). Only two PSHs (PSH 3 and PSH 4) produced more than one (two) *psb*A haplotypes that were separated by a single mutation (99.9% similarity).

Given the consistency between the two phylogenies, any PSH drawn using ABGD and GMYC that was either monophyletic for *psb*A or had unique *psb*A haplotypes was proposed as a SSH. As a result, 16 initial PSHs were converted to 13 SSHs (Fig. 2; see also Table S2b for average COI-5P sequence divergence between and within SSHs). Two pairs of PSHs alternatively recognized as either a single PSH or as two different PSHs by ABGD and GMYC were turned in a single SSH each (SSH 4+5, SSH 6+7). Unfortunately, we did not manage to obtain a *psb*A sequence for PSH 9, a PSH supported by only one of the partitions derived from the GMYC model. Since its COI-5P sequence was very close to the haplotypes found in PSH 8, we opted for a conservative inclusive approach and considered these two PSHs as a single SSH (8+9). The final partition into SSHs matched the delineation obtained with the single-threshold alternative of GMYC applied to COI-5P data only and was nearly identical to the less inclusive partition generated by ABGD.

### Matches in public data bases

Based on the literature and on the magnitude of intra-SSH variability found in our study, we used ad-hoc cutoff values (>98% identity for COI-5P, >99% for *psb*A) to determine which GenBank searches had returned hits for potential conspecifics. Only 9 out of our 29 COI-5P haplotypes (4 SSHs) and 3 out of the 15 *psb*A haplotypes (2 SSHs) resulted in a relevant match in either GenBank or BOLD (Table S3). Altogether, we obtained hits for 4 out of our 13 SSHs: SSH4+5, SSH6+7, SSH12, and SSH16. Only SSH4+5 resulted in a match to an identified species, *L. glaciale*: our COI-5P sequences were 98.6–99.9% similar to, and shared the same Barcode Index Number (BOLD:AAA6958), 39 accessions uploaded to BOLD for plants collected in Northeast USA and Canada. Also from OSPAR region I, SSH6+7 was conspecific with plants (BIN BOLD: ABA9580) from the Pacific (British Columbia) which, according to pictures logged in BOLD, have a branched morphology typical of maerl-forming plants. Finally, SSH12 and SSH16 had conspecific matches in GenBank with specimens from Brittany, France, which were only identified as Corallinales.

### Concordance with morphological identification

The total number of species identified based on their morphological features (10) was close to the number of SSHs (13) delimited with molecular data. However, among the 11 SSH with more than one specimen, only SSHs 1, 3, 11, and 13 were consistently assigned to a single morphospecies (Fig. 2). Many morphospecies contained collections from two, five or even six distinct SSHs. The only exceptions were *M. sphaericum*, a maerl species with a distinctive spherical morphology, and the three collections assigned to morphospecies *Lithothamnion* sp2 that clustered under SSH 2.

### Attribution of available species names

Six SSHs could be assigned to a species name using a body of proofs. In two cases, name assignment rested on comparisons with molecular data obtained from type material. SSH 12 was identical to COI-5P sequences obtained from neotype material of *P. calcareum* from BM. Likewise, our collections of SSH 1 included the holotype of *M. sphaericum*.

For the second most widespread and common species in our study (SSH 8+9), we tentatively attributed the species name *L. corallioides* because the latter, together with *P. calcareum*, is typically regarded as a common component of maerl beds in Atlantic Europe. Furthermore, samples from the type locality of *L. corallioides* (Rade de Brest, Finistère, France)[34] were resolved in the SSH 8+9.

In light of the morphological traits observed by SEM together with the existence of previous records from the same area, we temporarily attributed the names *L. fasciculatum* and *L. dentatum* to SSH 15 and SSH 16. In doing so, we used two names currently available in the literature for the European Atlantic while acknowledging that they belong to entities that need revision. A reassessment of the lectotype of *L. fasciculatum* has revealed that the epithet *fasciculatum* was misapplied to Atlantic plants belonging to the genus *Lithophyllum* [43]. Likewise, it seems unlikely that the coralline algae identified as *L. dentatum* in the Atlantic and their Mediterranean counterparts may be conspecifics [44]. Indeed, the Atlantic plants of *L. dentatum* were previously considered a form of *L. incrustans* Philippi [45].

Finally, we attributed the species name *L. glaciale* to SSH 4+5 based on the result of the BOLD identification engine. The name used in BOLD has not been confirmed by matching to sequences of type material (see [46]) and should be used with caution. Nonetheless, we also recorded SSH 4+5 in Spitsbergen Island (Svalbard Archipelago), the type locality of *L. glaciale* and where this coralline is reported to be common along the west and north coasts of the island (see [36] and references therein). We did not dare to link other SSHs to available species names and, therefore, seven SSHs were left without a binomial name. Nevertheless, their generic affiliations were evident based on their morphological traits and the phylogenetic relationships inferred from our COI-5P and *psb*A data, and we temporarily named them as *Lithothamnion* sp.2 (SSH 6+7), *Phymatolithon* sp.1 (SSH 10), *Phymatolithon* sp.2 (SSH 11), *Phymatolithon* sp.3 (SSH 13), *Phymatolithon* sp.4 (SSH 14), *Mesophyllum* sp.1 (SSH 2), and *Mesophyllum* sp.2 (SSH 3).

### Geographical distribution

**Figure 3:**
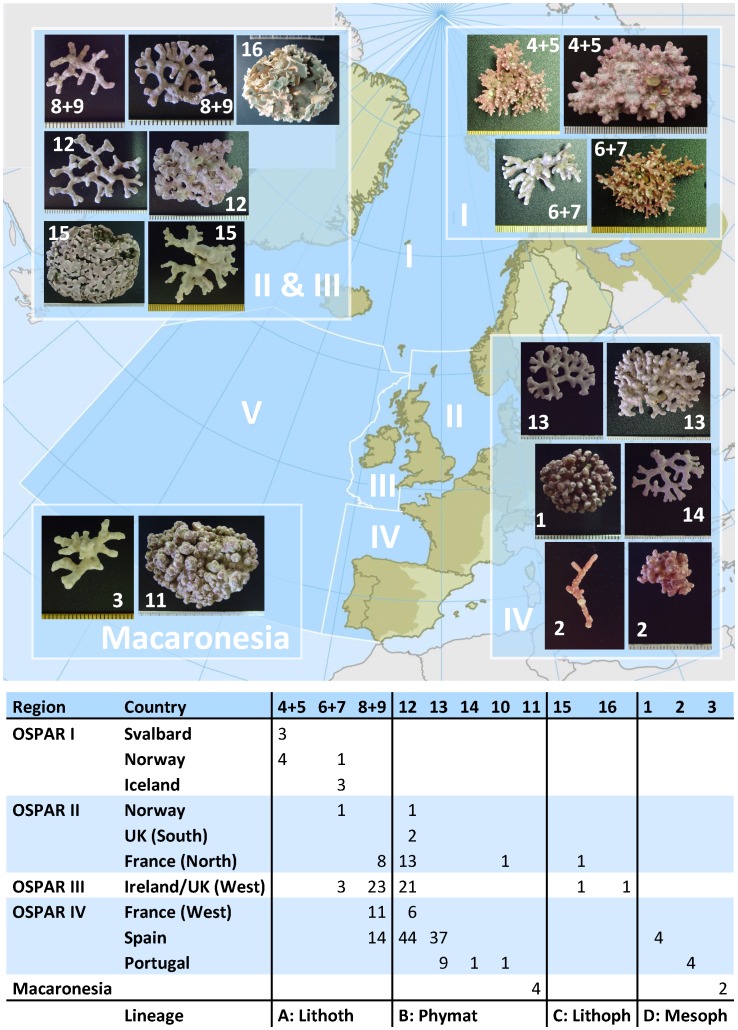
Morphological variability and distribution of the Secondary Species Hypotheses (SSHs) along the OSPAR area. The table indicates the number of specimens assigned to each SSH per OSPAR region and country. Vertical lines delimitate four major lineages revealed by both COI-5P and *psb*A phylogenies; tentative names are provided for the clades (genus level) based on sequence information from type/topotype material and on the occurrence of conspicuous morphological features. Lithoth.  =  *Lithothamnion*, Phymat.  =  *Phymatolithon*, Lithoph.  =  *Lithophyllum*, Mesoph.  =  *Mesophyllum*. Scale divisions in the photographs are mm.

A majority of SSHs were restricted to one (6 SSHs) or two (4 SSHs) sampling areas (Fig. 3). The remaining three SSHs showed wider, largely continuous distributions. *Lithothamnion* sp.2 (SSH 6+7; for equivalence between SSHs and species names see Fig. 2) seemed confined to high latitudes in OSPAR regions I and II where it showed minimal overlap with other maerl-forming plants except the phylogenetically close *L. glaciale* (SSH 4+5). Southward, *Lithothamnion* sp.2 was replaced by two species, *L. corallioides* (SSH 8+9) and *P. calcareum* (SSH 12), with wide ranges that reached northwest Spain. None of the species found in Macaronesia (Madeira and Canary Islands) was detected in the OSPAR area and the other way around.

Each major lineage resolved in our phylogeny had different ranges and limits. *Lithothamnion* (lineage A, Fig. 3) was detected at mid to high latitudes in the OSPAR area, with a southern edge in northwest Spain. *Phymatolithon* (Lineage B) went unrecorded in OSPAR region I; instead, it reached the warmer coasts of southern Portugal and Macaronesia, albeit with a replacement of species. Finally, *Lithophyllum* (lineage C) was restricted to the British Isles and north of France whereas *Mesophyllum* (lineage D) was confined to southern latitudes (Spain, Portugal and Macaronesia).

## Discussion

### Delimitation and identification of species of coralline algae forming maerl

Our spatially comprehensive sampling likely provides a thorough picture of the alpha diversity of maerl along European Atlantic coasts. Our analyses of both COI-5P and psbA sequence data have delineated 13 SSHs, a number comparable to the 16 species reported for the OSPAR regions and Macaronesia (see Table 1 for references). Nevertheless, linking available binomial names to the SSHs uncovered in our study was a delicate task. In addition to various analyses of the sequence data, additional evidence (morpho-anatomical observations, previously known species distribution, molecular data from type and topotype specimens) was required to guide our decisions at the time of attributing names. Following this approach, we managed to name almost half of the species detected in our study with acceptable confidence. However, it is likely that most, if not all, of the species that we left unnamed in this study may have already been described elsewhere.

We did not dare to identify SSH 6+7 (*Lithothamnion* sp. 2) to species level. Its confinement to OSPAR region I and to northernmost sites of region II suggests that *L. tophiforme* could be a plausible name but our sequences did not match two collections from New Foundland, Canada lodged in BOLD as *L. tophiforme*. However, the identity of these collections in BOLD has not been confirmed with sequence data from type material and should be considered with caution. *Lithothamion tophiforme* is mainly reported as an Arctic species that, in European waters, is confined to very high latitudes [47]. Somewhat unexpectedly, however, *L. tophiforme* went unrecorded in a detailed recent investigation of the northernmost maerl communities currently known, discovered in 2006 at 80°31’N in the Svalbard Archipelago [36]. Instead, these communities were dominated by *L. glaciale*, the only species that we found in our collections from Svalbard. Interestingly, our BOLD searches revealed that *Lithothamnion* sp. 2 also occurs in the North Pacific (British Columbia). A comparable circumpolar distribution has been reported for *L. glaciale* (see references in [48]).

Our results show that *Phymatolithon* sp.3 (SSH 13) is a major, even dominant, component of maerl beds in Spain and Portugal. Indeed, a recent quantitative study with DNA barcodes demonstrates that the widespread belief that *L. corallioides* and *P. calcareum* are the major builders of maerl in the temperate European Atlantic does not hold for the Iberian Peninsula. Instead, they are gradually replaced by *Phymatolithon* sp.3 in Galicia (NW Spain) to become extremely rare in S Portugal [49]. Despite our efforts, we have been unable to resolve the identity of this species beyond generic level. The examination by SEM revealed traits also found in *Phymatolithon lamii* (e.g. sunken, rimless conceptacles), a common coralline throughout the British Isles, northern Spain, France, Norway, Iceland and eastern North America [50]. It has also been reported from the western North Pacific Ocean [51], [52] and, more recently, from the Mediterranean where it might be an alien species [53]. In addition, one of the co-authors (V.P., unpublished results) recently sequenced a 600 bp long fragment of the *psb*A gene from the type specimen of *P. lamii* (in PC) that reveals a low-moderate divergence with our *Phymatolithon* sp.3. However, *P. lamii* has always been described as encrusting thalli, and there is no previous record of its occurrence as maerl. Hence, further sequence data from type material will be required to assign a species name to SSH13. Lastly, our results clearly indicate that the maerl-forming algae that colonize Macaronesia deserve further study with appropriate sampling design and molecular tools.

To our knowledge, this is the first barcoding study focused on maerl-forming algae. Previous DNA barcode studies on coralline algae mostly focused on geniculate forms [13], [22], [46], [54] or were intended to resolve infra-ordinal phylogenetic relationships among the Corallinales [55]. Nevertheless, Bittner *et al.* [56] sequenced geniculated and non-geniculated coralline algae, mainly from South Pacific Islands, for *psb*A and COI-5P, and used ABGD and GYMC to delineate species. As the authors found very divergent numbers of ‘genetic species’ depending on the criteria and the marker, they concluded that DNA-barcoding was non-accurate for assessing the species diversity in this group (but see [33] for alternative explanations when GMYC has a highly divergent outcome). Contrarily, we are in favor of an integrative systematic approach to investigate the diversity of maerl-forming red algae. We propose the use of the mitochondrial COI-5P barcode as the first marker and the plastidial *psb*A gene as a secondary marker along with other lines of evidence. In this regard, we follow other authors that already noted the intrinsic limitations of delimiting species from single-locus studies and advocate the incorporation of multiple lines of evidence (biogeographical, biological, additional gene sequences) in this studies [10], [32], [33].

Despite considerable efforts to identify specimens collected in this study based on their morpho-anatomical characters, sequence data were incomparably more efficient. In fact, our Fig. 2 shows that even maerl assigned to different morphogenera turned out to belong to the same molecular entity. Initially, this considerable discrepancy between the morphological identification and the molecular-based delimitation might seem astonishing. However, it is just another example of the considerable challenge of discriminating species in a group that largely lacks diagnostic features but shows considerable morphological plasticity and convergence. Species delimitation with molecular markers is known to perform well with deeply divergent taxa such as those found in our study. However, we still uncovered a few closely related species that deserve further studies. It is increasingly acknowledged that a multi-locus approach can define reliable species boundaries in those cases [10], [29], [31], [57], [58]. Even for the more closely related SSHs, the genealogical concordance observed between loci located in different genomes lends additional support to our partition. Finally, the congruence between partitions recovered with analyses based on radically different criteria (coalescence vs. distribution of pairwise differences between sequences) indicates that there is a strong signal in the COI-5P data set. If any, the only shortcoming encountered in the course of that study was the erratic amplification of the COI-5P fragment that failed on 1/3 of the specimens at the first attempt, although repeated amplifications and/or re-extracting DNA from the same individual often solved this issue. The remarkable coincidence between the patterns revealed by our *psb*A and COI-5P sequence data indicates that the plastidial marker, which is easier to amplify than COI-5P, could be a useful alternative for the identification of maerl-forming species (but see [54] for a intrageneric study where the gene trees produced by COI-5P and by another plastid-encoded gene, *rbc*L, were not always congruent). Compared to the standard DNA barcode, *psb*A has lower resolution. However, most of the maerl-forming taxa studied in the OSPAR region are deeply divergent. The only exceptions were two pairs of closely related species with low levels of interspecific divergence (2–3 nucleotide substitutions) that could be mistaken for intraspecific variability (*L. fasciculatum vs. L. dentatum*; *Phymatolithon* sp.3 vs. *Phymatolithon* sp.4).

### Species distribution and implication for future prospects

Our study reveals that two species of coralline algae are the main constituent of maerl beds in temperate European Atlantic: *L. corallioides* and *P. calcareum*. Another *Phymatolithon* (SSH13) replaces them in the south while the cold OSPAR region I seems dominated by two species of *Lithothamnion* (*L. glaciale* and *Lithothamnion* sp.2-SSH 6+7). The remaining species unraveled in our study are either infrequent and/or confined in space. The gradual replacement of species with latitude (Fig. 3) is consistent with the patterns of maerl distribution reported in the literature (see Table 1 and references therein). The distribution of coralline algae forming maerl in our study is likewise consistent with a general pattern observed in many taxonomic groups where a majority of species have small geographic ranges whereas a few have large ones [59]. Biogeographical distribution patterns of species are strongly controlled by climate [60], [61]. For instance, the confinement of *L. glaciale* to arctic and subarctic locations has been attributed to the fact that this plant only produces reproductive conceptacles when water temperatures are below 9°C in winter [62]. Maerl forming coralline algae are likely to be affected by the ongoing global warming [63]. They may migrate to regions where the climatic conditions are suitable for their physiology or may become extinct. In that context, our study provides an assessment of genuine distribution of maerl species as well as an efficient tool to monitor putative shifts in southern and northern ranges of each species delineated.

## Supporting Information

Table S1 Collection details with BOLD IDs and GenBank accession numbers for samples used in this study.
(DOCX)

Table S2 **A.** COI-5P sequence divergence within (diagonal) and between (below diagonal) the PSHs shown in Fig. 1. **B.** COI-5P sequence divergence within (diagonal) and between (below diagonal) the SSHs shown in Fig. 2.
(PDF)

Table S3 Matches with our sequences in public databases (Genbank: COI-5P and *psb*A; BOLD: COI-5P).
(PDF)
