# Supplementary material for: A Multilocus Species Delimitation Reveals a Striking Number of Species of Coralline Algae Forming Maerl in the OSPAR Maritime Area
Source: PLoS One. 2014 Aug 11;9(8):e104073. doi: 10.1371/journal.pone.0104073 (PMC4128821; doi:10.1371/journal.pone.0104073)
Supplement: Table S1 — Collection details with BOLD IDs and GenBank accession numbers for samples used in this study. (DOCX) [file pone.0104073.s001.docx]

**Table S1.** **Collection details with BOLD IDs and GenBank accession numbers for samples used in this study.**

| **Taxon** | **Voucher**^1^ | **Collectors**^2^ | **Date** | **Haplotype** | **Country** | **Lat** | **Long** | **Depth (m)** | **BOLD ID**^3, 4^ | **GenBank**^5^ |
| --- | --- | --- | --- | --- | --- | --- | --- | --- | --- | --- |
| *Lithophyllum fasciculatum* | CPVP-965 | JG | 01-Apr-11 | COI hap_16 / *psb*A hap_5 | France (Brittany) | 48.327 | -4.319 | 1 | **MAERL096-11** | KC861444  (KC819267) |
|  | CPVP-303 | JHS | 22-Feb-99 | COI hap_16 / *psb*A hap_5 | Ireland (Galway) | 53.471 | -10.117 | 3 | **MAERL171-13** | KC861445  (KC819247) |
| *Lithophyllum dentatum* | CPVP-302 | JHS | 22-Feb-99 | COI hap_18 / *psb*A hap_4 | Ireland (Galway) | 53.471 | -10.117 | 3 | **MAERL213-13** | KC861446  (KC819246) |
| *Lithothamnion corallioides* | CPVP-802 | JG | 08-Mar-11 | COI hap_4 | France (Brittany) | 48.207 | -4.452 | 10 | MAERL017-11 | KC861447 |
|  | CPVP-817 | JG | 06-Mar-11 | COI hap_3 / *psb*A hap_12 | France (Brittany) | 47.331 | -3.132 | 5 | **MAERL097-11** | KC861448  ( KC819265) |
|  | CPVP-803 | JG | 08-Mar-11 | COI hap_3 | France (Brittany) | 48.212 | -4.455 | 10 | MAERL181-13 | KC861449 |
|  | **CPVP-807*** | JG | 08-Mar-11 | COI hap_3 | France (Brittany) | 48.293 | -4.579 | 15 | MAERL179-13 | KC861451 |
|  | **CPVP-808*** | JG | 08-Mar-11 | COI hap_3 / *psb*A hap_12 | France (Brittany) | 48.293 | -4.579 | 15 | **MAERL178-13** | KC861452  (KC819264) |
|  | **CPVP-813*** | JG | 08-Mar-11 | COI hap_3 | France (Brittany) | 48.293 | -4.579 | 15 | MAERL027-11 | KC861465 |
|  | CPVP-820 | JG | 06-Mar-11 | COI hap_3 | France (Brittany) | 47.331 | -3.132 | 5 | MAERL177-13 | KC861453 |
|  | CPVP-823 | JG | 06-Mar-11 | COI hap_3 | France (Brittany) | 47.331 | -3.132 | 5 | MAERL176-13 | KC861454 |
|  | CPVP-825 | JG | 06-Mar-11 | COI hap_3 | France (Brittany) | 47.331 | -3.132 | 5 | MAERL175-13 | KC861455 |
|  | CPVP-819 | JG | 06-Mar-11 | COI hap_3 | France (Brittany) | 47.331 | -3.132 | 5 | MAERL030-11 | KC861462 |
|  | CPVP-816 | JG | 06-Mar-11 | COI hap_3 | France (Brittany) | 47.331 | -3.132 | 5 | MAERL029-11 | KC861463 |
|  | CPVP-818 | JG | 06-Mar-11 | COI hap_3 | France (Brittany) | 47.331 | -3.132 | 5 | MAERL028-11 | KC861464 |
|  | CPVP-801 | JG | 08-Mar-11 | COI hap_3 | France (Brittany) | 48.207 | -4.452 | 10 | MAERL021-11 | KC861471 |
|  | CPVP-799 | JG | 08-Mar-11 | COI hap_3 | France (Brittany) | 48.212 | -4.455 | 10 | MAERL183-13 | KC861502 |
|  | CPVP-794 | JG | 08-Mar-11 | COI hap_3 | France (Brittany) | 48.212 | -4.455 | 10 | MAERL184-13 | KC861501 |
|  | CPVP-1238 | JJ | 22-Jul-11 | COI hap_4 | France (La Rochelle) | 46.233 | -1.381 | 12 | MAERL188-13 | KC861497 |
|  | CPVP-1227 | JJ | 22-Jul-11 | COI hap_3 | France (La Rochelle) | 46.233 | -1.381 | 12 | MAERL187-13 | KC861498 |
|  | CPVP-1231 | JJ | 22-Jul-11 | COI hap_4 | France (La Rochelle) | 46.233 | -1.381 | 12 | MAERL186-13 | KC861499 |
|  | CPVP-1232 | JJ | 22-Jul-11 | COI hap_4 | France (La Rochelle) | 46.233 | -1.381 | 12 | MAERL185-13 | KC861500 |
|  | CPVP-166 | IB, RB, CP, VP | 23-Nov-10 | COI hap_3 | Spain (Galicia) | 42.561 | -8.890 | 6 | MAERL180-13 | KC861450 |
|  | CPVP-1077 | IB, FB, VP | 23-Jun-11 | COI hap_3 | Spain (Galicia) | 42.569 | -8.890 | 4 | MAERL174-13 | KC861456 |
|  | CPVP-1079 | IB, FB, VP | 23-Jun-11 | COI hap_3 | Spain (Galicia) | 42.569 | -8.890 | 4 | MAERL173-13 | KC861457 |
|  | CPVP-649 | IB, RB, VP | 05-Apr-11 | COI hap_3 | Spain (Galicia) | 42.212 | -8.896 | 11 | MAERL034-11 | KC861458 |
|  | CPVP-564 | IB, RB, CP, VP | 31-Mar-11 | COI hap_3 | Spain (Galicia) | 42.788 | -9.019 | 11 | MAERL033-11 | KC861459 |
|  | CPVP-563 | IB, RB, CP, VP | 31-Mar-11 | COI hap_3 / *psb*A hap_12 | Spain (Galicia) | 42.788 | -9.019 | 11 | **MAERL032-11** | KC861460  (KC819256) |
|  | CPVP-561 | IB, RB, CP, VP | 31-Mar-11 | COI hap_3 | Spain (Galicia) | 42.788 | -9.019 | 11 | MAERL031-11 | KC861461 |
|  | CPVP-684 | IB, RB, VP | 05-Apr-11 | COI hap_3 | Spain (Galicia) | 42.258 | -8.751 | 5 | MAERL026-11 | KC861466 |
|  | CPVP-691 | IB, RB, VP | 05-Apr-11 | COI hap_3 / *psb*A hap_12 | Spain (Galicia) | 42.258 | -8.751 | 5 | **MAERL025-11** | KC861467  (KC819261) |
|  | CPVP-697 | IB, RB, VP | 05-Apr-11 | COI hap_3 | Spain (Galicia) | 42.258 | -8.751 | 5 | MAERL024-11 | KC861468 |
|  | CPVP-677 | IB, RB, VP | 05-Apr-11 | COI hap_3 | Spain (Galicia) | 42.258 | -8.751 | 5 | MAERL023-11 | KC861469 |
|  | CPVP-699 | IB, RB, VP | 05-Apr-11 | COI hap_3 | Spain (Galicia) | 42.258 | -8.751 | 5 | MAERL022-11 | KC861470 |
|  | CPVP-138 | IB, RB, CP, VP | 23-Nov-10 | COI hap_3 | Spain (Galicia) | 42.569 | -8.890 | 4 | MAERL018-11 | KC861472 |
|  | CPVP-631 | IB, RB, VP | 07-Apr-11 | COI hap_3 | Spain (Galicia) | 42.341 | -8.810 | 9 | MAERL211-13 | KC861474 |
|  | CPVP-1250 | LK, DP | 25-Jul-11 | COI hap_4 | UK (Northern Ireland) | 54.378 | -5.565 | 10 | MAERL212-13 | KC861473 |
|  | CPVP-1164 | FB | 14-Jul-11 | COI hap_3 | UK (Wales) | 51.709 | -5.085 | 4 | MAERL210-13 | KC861475 |
|  | CPVP-1184 | FB | 14-Jul-11 | COI hap_3 | UK (Wales) | 51.709 | -5.085 | 4 | MAERL209-13 | KC861476 |
|  | CPVP-1189 | FB | 14-Jul-11 | COI hap_3 | UK (Wales) | 51.709 | -5.085 | 4 | MAERL208-13 | KC861477 |
|  | CPVP-1191 | FB | 14-Jul-11 | COI hap_3 | UK (Wales) | 51.709 | -5.085 | 4 | MAERL207-13 | KC861478 |
|  | CPVP-1192 | FB | 14-Jul-11 | COI hap_3 | UK (Wales) | 51.709 | -5.085 | 4 | MAERL206-13 | KC861479 |
|  | CPVP-1193 | FB | 14-Jul-11 | COI hap_3 | UK (Wales) | 51.709 | -5.085 | 4 | MAERL205-13 | KC861480 |
|  | CPVP-1196 | FB | 14-Jul-11 | COI hap_3 | UK (Wales) | 51.709 | -5.085 | 4 | MAERL204-13 | KC861481 |
|  | CPVP-1199 | FB | 14-Jul-11 | COI hap_3 | UK (Wales) | 51.709 | -5.085 | 4 | MAERL203-13 | KC861482 |
|  | CPVP-1201 | FB | 14-Jul-11 | COI hap_3 | UK (Wales) | 51.709 | -5.085 | 4 | MAERL202-13 | KC861483 |
|  | CPVP-1163 | FB | 14-Jul-11 | COI hap_3 | UK (Wales) | 51.709 | -5.085 | 4 | MAERL201-13 | KC861484 |
|  | CPVP-1165 | FB | 14-Jul-11 | COI hap_3 | UK (Wales) | 51.709 | -5.085 | 4 | MAERL200-13 | KC861485 |
|  | CPVP-1166 | FB | 14-Jul-11 | COI hap_3 | UK (Wales) | 51.709 | -5.085 | 4 | MAERL199-13 | KC861486 |
|  | CPVP-1167 | FB | 14-Jul-11 | COI hap_25 | UK (Wales) | 51.709 | -5.085 | 4 | MAERL198-13 | KC861487 |
|  | CPVP-1171 | FB | 14-Jul-11 | COI hap_3 | UK (Wales) | 51.709 | -5.085 | 4 | MAERL197-13 | KC861488 |
|  | CPVP-1172 | FB | 14-Jul-11 | COI hap_3 | UK (Wales) | 51.709 | -5.085 | 4 | MAERL196-13 | KC861489 |
|  | CPVP-1173 | FB | 14-Jul-11 | COI hap_3 | UK (Wales) | 51.709 | -5.085 | 4 | MAERL195-13 | KC861490 |
|  | CPVP-1174 | FB | 14-Jul-11 | COI hap_3 | UK (Wales) | 51.709 | -5.085 | 4 | MAERL194-13 | KC861491 |
|  | CPVP-1176 | FB | 14-Jul-11 | COI hap_3 | UK (Wales) | 51.709 | -5.085 | 4 | MAERL193-13 | KC861492 |
|  | CPVP-1179 | FB | 14-Jul-11 | COI hap_3 | UK (Wales) | 51.709 | -5.085 | 4 | MAERL192-13 | KC861493 |
|  | CPVP-1181 | FB | 14-Jul-11 | COI hap_3 | UK (Wales) | 51.709 | -5.085 | 4 | MAERL191-13 | KC861494 |
|  | CPVP-1182 | FB | 14-Jul-11 | COI hap_3 | UK (Wales) | 51.709 | -5.085 | 4 | MAERL190-13 | KC861495 |
|  | CPVP-1183 | FB | 14-Jul-11 | COI hap_3 | UK (Wales) | 51.709 | -5.085 | 4 | MAERL189-13 | KC861496 |
| *Lithothamnion glaciale* | CPVP-93 | VH | 01-May-08 | COI hap_7 | Norway (Skarsundet) | 60.143 | 5.166 | 11 | MAERL229-13 | KC861503 |
|  | CPVP-1401 | VH | 01-May-08 | COI hap_7 | Norway (Skarsundet) | 60.143 | 5.166 | 11 | MAERL228-13 | KC861504 |
|  | CPVP-1403 | VH | 01-May-08 | COI hap_7 | Norway (Skarsundet) | 60.143 | 5.166 | 11 | MAERL227-13 | KC861505 |
|  | CPVP-91 | VH | 01-May-08 | COI hap_7 / *psb*A hap_2 | Norway (Skarsundet) | 60.143 | 5.166 | 11 | **MAERL230-13** | KC861508  (KC819244) |
|  | **CPVP-1448*** | JB | 01-Jun-10 | COI hap_7 | Norway (Svalbard) | 78.955 | 9.668 | 10 | MAERL226-13 | KC861506 |
|  | **CPVP-1443*** | JB | 01-Jun-10 | COI hap_24 / *psb*A hap_15 | Norway (Svalbard) | 78.955 | 9.668 | 10 | **MAERL225-13** | KC861507  (KC819270) |
|  | **CPVP-1444*** | JB | 01-Jun-10 | COI hap_7 / *psb*A hap_15 | Norway (Svalbard) | 78.955 | 9.668 | 10 | **MAERL224-13** | KC861509  (KC819271) |
| *Lithothamnion* sp. 1 | CPVP-30 | VP, HH, ARE | 20-Jul-04 | COI hap_17 | Iceland (Hvalfjoerdur) | 64.360 | -21.753 | 4 | MAERL218-13 | KC861510 |
|  | CPVP-28 | VP, HH, ARE | 20-Jul-04 | COI hap_17 | Iceland (Hvalfjoerdur) | 64.360 | -21.753 | 4 | MAERL219-13 | KC861515 |
|  | CPVP-29 | VP, HH, ARE | 20-Jul-04 | COI hap_19 | Iceland (Hvalfjordur) | 64.360 | -21.753 | 3 | MAERL035-11 | KC861516 |
|  | CPVP-92 | VH | 01-May-08 | COI hap_5 / *psb*A hap_3 | Norway (Skarsundet) | 60.143 | 5.166 | 11 | **MAERL222-13** | KC861512  (KC819245) |
|  | CPVP-1451 | JR | 01-Jan-02 | COI hap_5 | Norway (Hordaland) | 60.541 | 4.846 | 7 | MAERL221-13 | KC861513 |
|  | CPVP-1240 | LK, DP | 25-Jul-11 | COI hap_17 | UK (Northern Ireland) | 54.378 | -5.565 | 10 | MAERL223-13 | KC861511 |
|  | CPVP-306 | NK | 29-Oct-10 | COI hap_17 | UK (Scotland) | 56.022 | -5.611 | 7 | MAERL220-13 | KC861514 |
|  | CPVP-305 | NK | 29-Oct-10 | COI hap_17 / *psb*A hap_3 | UK (Scotland) | 56.022 | -5.611 | 7 | **MAERL011-11** | KC861517  (KC819248) |
| *Mesophyllum* sp. 1 | CPVP-514 | PN, MR | 23-Feb-11 | COI hap_11 | Portugal (Algarve) | 37.046 | -8.336 | 20 | MAERL014-11 | KC861518 |
|  | CPVP-464 | PN, MR | 02-Mar-11 | COI hap_11 / *psb*A hap_8 | Portugal (Algarve) | 37.027 | -8.317 | 20 | **MAERL019-11** | KC861519  (KC819252) |
|  | CPVP-465 | PN, MR | 02-Mar-11 | COI hap_11 | Portugal (Algarve) | 37.027 | -8.317 | 20 | MAERL013-11 | KC861520 |
|  | CPVP-467 | PN, MR | 02-Mar-11 | COI hap_11 | Portugal (Algarve) | 37.027 | -8.317 | 20 | MAERL020-11 | KC861521 |
| *Mesophyllum* sp. 2 | CPVP-1157 | CS | 15-Apr-10 | COI hap_27 / *psb*A hap_14 | Spain (Canary Islands) | 28.642 | -17.723 | 15 | **MAERL217-13** | KC861522  (KC819269) |
|  | CPVP-307 | CS | 15-Apr-10 | COI hap_27 / *psb*A hap_6 | Spain (Canary Islands) | 28.642 | -17.723 | 15 | **MAERL016-11** | KC861523  (KC819249) |
| *Mesophyllum sphaericum* | **CPVP-1084*** | IB, FB, VP | 23-Jun-11 | COI hap_2 | Spain (Galicia) | 42.600 | -8.874 | 3 | MAERL216-13 | KC861524 |
|  | **CPVP-1130*** | IB, FB, VP | 23-Jun-11 | COI hap_2 / *psb*A hap_13 | Spain (Galicia) | 42.600 | -8.874 | 3 | **MAERL214-13** | KC861525  (KC819268) |
|  | **CPVP-776**** | IB, VP | 14-Oct-08 | COI hap_2 / *psb*A hap_13 | Spain (Galicia) | 42.600 | -8.874 | 3 | **MAERL015-11** | KC861526  (KC819262) |
|  | **CPVP-1115*** | IB, FB, VP | 23-Jun-11 | COI hap_6 | Spain (Galicia) | 42.600 | -8.874 | 3 | MAERL215-13 | KC861527 |
| *Phymatolithon calcareum* | CPVP-555 | IB, RB, CP, VP | 31-Mar-11 | COI hap_26 | Spain (Galicia) | 42.788 | -9.019 | 11 | MAERL041-11 | KC861528 |
|  | CPVP-753 | VP, IB, RB | 05-Apr-11 | COI hap_26 | Spain (Galicia) | 42.227 | -8.777 | 11 | MAERL136-13 | KC861546 |
|  | CPVP-603 | IB, RB, VP | 07-Apr-11 | COI hap_26 | Spain (Galicia) | 42.394 | -8.915 | 13 | MAERL132-13 | KC861550 |
|  | CPVP-607 | IB, RB, VP | 07-Apr-11 | COI hap_28 | Spain (Galicia) | 42.394 | -8.915 | 13 | MAERL131-13 | KC861551 |
|  | CPVP-615 | IB, RB, VP | 07-Apr-11 | COI hap_26 | Spain (Galicia) | 42.394 | -8.915 | 13 | MAERL130-13 | KC861552 |
|  | CPVP-162 | IB, RB, CP, VP | 23-Nov-10 | COI hap_26 | Spain (Galicia) | 42.561 | -8.890 | 6 | MAERL117-13 | KC861565 |
|  | CPVP-163 | IB, RB, CP, VP | 23-Nov-10 | COI hap_26 | Spain (Galicia) | 42.561 | -8.890 | 6 | MAERL116-13 | KC861566 |
|  | CPVP-164 | IB, RB, CP, VP | 23-Nov-10 | COI hap_26 | Spain (Galicia) | 42.561 | -8.890 | 6 | MAERL115-13 | KC861567 |
|  | CPVP-170 | IB, RB, CP, VP | 23-Nov-10 | COI hap_26 | Spain (Galicia) | 42.561 | -8.890 | 6 | MAERL114-13 | KC861568 |
|  | CPVP-655 | IB, RB, VP | 05-Apr-11 | COI hap_26 | Spain (Galicia) | 42.212 | -8.896 | 11 | MAERL113-13 | KC861569 |
|  | CPVP-1103 | IB, FB, VP | 23-Jun-11 | COI hap_26 | Spain (Galicia) | 42.600 | -8.874 | 3 | MAERL112-13 | KC861570 |
|  | CPVP-1104 | IB, FB, VP | 23-Jun-11 | COI hap_26 | Spain (Galicia) | 42.600 | -8.874 | 3 | MAERL111-13 | KC861571 |
|  | CPVP-1108 | IB, FB, VP | 23-Jun-11 | COI hap_26 | Spain (Galicia) | 42.600 | -8.874 | 3 | MAERL110-13 | KC861572 |
|  | CPVP-1109 | IB, FB, VP | 23-Jun-11 | COI hap_26 | Spain (Galicia) | 42.600 | -8.874 | 3 | MAERL109-13 | KC861573 |
|  | CPVP-1110 | IB, FB, VP | 23-Jun-11 | COI hap_26 | Spain (Galicia) | 42.600 | -8.874 | 3 | MAERL108-13 | KC861574 |
|  | CPVP-1118 | IB, FB, VP | 23-Jun-11 | COI hap_26 | Spain (Galicia) | 42.600 | -8.874 | 3 | MAERL107-13 | KC861575 |
|  | CPVP-1119 | IB, FB, VP | 23-Jun-11 | COI hap_26 | Spain (Galicia) | 42.600 | -8.874 | 3 | MAERL106-13 | KC861576 |
|  | CPVP-1120 | IB, FB, VP | 23-Jun-11 | COI hap_26 | Spain (Galicia) | 42.600 | -8.874 | 3 | MAERL105-13 | KC861577 |
|  | CPVP-1121 | IB, FB, VP | 23-Jun-11 | COI hap_26 | Spain (Galicia) | 42.600 | -8.874 | 3 | MAERL104-13 | KC861578 |
|  | CPVP-1129 | IB, FB, VP | 23-Jun-11 | COI hap_26 | Spain (Galicia) | 42.600 | -8.874 | 3 | MAERL103-13 | KC861579 |
|  | CPVP-1065 | IB, FB, VP | 23-Jun-11 | COI hap_26 | Spain (Galicia) | 42.569 | -8.890 | 5 | MAERL102-13 | KC861580 |
|  | CPVP-1067 | IB, FB, VP | 23-Jun-11 | COI hap_26 | Spain (Galicia) | 42.569 | -8.890 | 5 | MAERL101-13 | KC861581 |
|  | CPVP-1078 | IB, FB, VP | 13-Jun-11 | COI hap_26 | Spain (Galicia) | 42.569 | -8.890 | 5 | MAERL100-13 | KC861582 |
|  | CPVP-174 | IB, RB, CP, VP | 23-Nov-10 | COI hap_26 | Spain (Galicia) | 42.569 | -8.890 | 4 | MAERL099-13 | KC861583 |
|  | CPVP-207 | IB, RB, CP, VP | 23-Nov-10 | COI hap_26 | Spain (Galicia) | 42.569 | -8.890 | 4 | MAERL098-13 | KC861584 |
|  | CPVP-628 | IB, RB, VP | 07-Apr-11 | COI hap_26 | Spain (Galicia) | 42.341 | -8.810 | 9 | MAERL036-11 | KC861586 |
|  | CPVP-629 | IB, RB, VP | 07-Apr-11 | COI hap_26 | Spain (Galicia) | 42.341 | -8.810 | 9 | MAERL037-11 | KC861587 |
|  | CPVP-558 | IB, RB, CP, VP | 31-Mar-11 | COI hap_26 | Spain (Galicia) | 42.788 | -9.019 | 11 | MAERL040-11 | KC861590 |
|  | CPVP-560 | IB, RB, CP, VP | 31-Mar-11 | COI hap_26 | Spain (Galicia) | 42.788 | -9.019 | 11 | MAERL042-11 | KC861592 |
|  | CPVP-554 | IB, RB, CP, VP | 31-Mar-11 | COI hap_26 | Spain (Galicia) | 42.788 | -9.019 | 11 | MAERL043-11 | KC861593 |
|  | CPVP-310 | IB, VP | 12-Jun-09 | COI hap_26 | Spain (Galicia) | 42.788 | -9.019 | 11 | MAERL044-11 | KC861594 |
|  | CPVP-566 | IB, RB, CP, VP | 31-Mar-11 | COI hap_26 / *psb*A hap_1 | Spain (Galicia) | 42.788 | -9.019 | 11 | **MAERL045-11** | KC861595  (KC819257) |
|  | CPVP-565 | IB, RB, CP, VP | 31-Mar-11 | COI hap_26 | Spain (Galicia) | 42.788 | -9.019 | 11 | MAERL046-11 | KC861596 |
|  | CPVP-766 | IB, RB, VP | 07-Apr-11 | COI hap_26 | Spain (Galicia) | 42.394 | -8.915 | 13 | MAERL047-11 | KC861597 |
|  | CPVP-167 | IB, RB, CP, VP | 23-Nov-10 | COI hap_26 | Spain (Galicia) | 42.561 | -8.890 | 6 | MAERL048-11 | KC861598 |
|  | CPVP-665 | IB, RB, VP | 05-Apr-11 | COI hap_26 / *psb*A hap_1 | Spain (Galicia) | 42.212 | -8.896 | 11 | **MAERL049-11** | KC861599  (KC819259) |
|  | CPVP-648 | IB, RB, VP | 05-Apr-11 | COI hap_26 | Spain (Galicia) | 42.212 | -8.896 | 11 | MAERL050-11 | KC861600 |
|  | CPVP-654 | IB, RB, VP | 05-Apr-11 | COI hap_26 | Spain (Galicia) | 42.212 | -8.896 | 11 | MAERL051-11 | KC861601 |
|  | CPVP-696 | IB, RB, VP | 05-Apr-11 | COI hap_26 | Spain (Galicia) | 42.258 | -8.751 | 5 | MAERL053-11 | KC861603 |
|  | CPVP-275 | IB, RB, CP, VP | 23-Nov-10 | COI hap_26 | Spain (Galicia) | 42.569 | -8.890 | 4 | MAERL061-11 | KC861611 |
|  | CPVP-130 | IB, RB, CP, VP | 23-Nov-10 | COI hap_26 | Spain (Galicia) | 42.569 | -8.890 | 4 | MAERL062-11 | KC861612 |
|  | CPVP-135 | IB, RB, CP, VP | 23-Nov-10 | COI hap_26 | Spain (Galicia) | 42.569 | -8.890 | 4 | MAERL063-11 | KC861613 |
|  | CPVP-151 | IB, RB, CP, VP | 23-Nov-10 | COI hap_26 | Spain (Galicia) | 42.569 | -8.890 | 4 | MAERL064-11 | KC861614 |
|  | CPVP-157 | IB, RB, CP, VP | 23-Nov-10 | COI hap_26 | Spain (Galicia) | 42.569 | -8.890 | 4 | MAERL065-11 | KC861615 |
|  | CPVP-1450 | JR | 01-Jan-02 | COI hap_26 | Norway (Hordaland) | 60.541 | 4.846 | 7 | MAERL133-13 | KC861549 |
|  | CPVP-1242 | LK, DP | 25-Jul-11 | COI hap_26 | UK (Northern Ireland) | 54.378 | -5.565 | 10 | MAERL149-13 | KC861533 |
|  | CPVP-1243 | LK, DP | 25-Jul-11 | COI hap_26 | UK (Northern Ireland) | 54.378 | -5.565 | 10 | MAERL148-13 | KC861534 |
|  | CPVP-1244 | LK, DP | 25-Jul-11 | COI hap_26 | UK (Northern Ireland) | 54.378 | -5.565 | 10 | MAERL147-13 | KC861535 |
|  | CPVP-1245 | LK, DP | 25-Jul-11 | COI hap_26 | UK (Northern Ireland) | 54.378 | -5.565 | 10 | MAERL146-13 | KC861536 |
|  | CPVP-1246 | LK, DP | 25-Jul-11 | COI hap_26 | UK (Northern Ireland) | 54.378 | -5.565 | 10 | MAERL145-13 | KC861537 |
|  | CPVP-1247 | LK, DP | 25-Jul-11 | COI hap_26 | UK (Northern Ireland) | 54.378 | -5.565 | 10 | MAERL144-13 | KC861538 |
|  | CPVP-1251 | LK, DP | 25-Jul-11 | COI hap_26 | UK (Northern Ireland) | 54.378 | -5.565 | 10 | MAERL143-13 | KC861539 |
|  | CPVP-1252 | LK, DP | 25-Jul-11 | COI hap_26 | UK (Northern Ireland) | 54.378 | -5.565 | 10 | MAERL142-13 | KC861540 |
|  | CPVP-1253 | LK, DP | 25-Jul-11 | COI hap_26 | UK (Northern Ireland) | 54.378 | -5.565 | 10 | MAERL141-13 | KC861541 |
|  | CPVP-1254 | LK, DP | 25-Jul-11 | COI hap_26 | UK (Northern Ireland) | 54.378 | -5.565 | 10 | MAERL140-13 | KC861542 |
|  | CPVP-43 | KC, VP | 07-May-05 | COI hap_29 | UK (England) | 50.605 | -1.868 | 12 | MAERL135-13 | KC861547 |
|  | CPVP-44 | KC, VP | 07-May-05 | COI hap_26 | UK (England) | 50.605 | -1.868 | 12 | MAERL134-13 | KC861548 |
|  | **CPVP-46*** | JHS | 02-Jun-10 | COI hap_26 | UK (England) | 50.164 | -5.022 | 18 | MAERL118-13 | KC861564 |
|  | **CPVP-48*** | JHS | 02-Jun-10 | COI hap_26 | UK (England) | 50.164 | -5.022 | 18 | MAERL012-11 | KC861585 |
|  | **CPVP-47*** | JHS | 02-Jun-10 | COI hap_26 / *psb*A hap_1 | UK (England) | 50.164 | -5.022 | 18 | **MAERL054-11** | KC861604  (KC819243) |
|  | CPVP-1187 | FB | 14-Jul-11 | COI hap_26 | UK (Wales) | 51.709 | -5.085 | 4 | MAERL126-13 | KC861556 |
|  | CPVP-1195 | FB | 14-Jul-11 | COI hap_26 | UK (Wales) | 51.709 | -5.085 | 4 | MAERL125-13 | KC861557 |
|  | CPVP-1188 | FB | 14-Jul-11 | COI hap_26 | UK (Wales) | 51.709 | -5.085 | 4 | MAERL124-13 | KC861558 |
|  | CPVP-780 | MM, JH | 21-Oct-10 | COI hap_26 | Ireland (Galway) | 53.246 | -9.628 | 5 | MAERL120-13 | KC861562 |
|  | CPVP-783 | MM, JH | 21-Oct-10 | COI hap_26 / *psb*A hap_1 | Ireland (Galway) | 53.246 | -9.628 | 5 | **MAERL119-13** | KC861563  (KC819263) |
|  | CPVP-781 | MM, JH | 21-Oct-10 | COI hap_26 | Ireland (Galway) | 53.246 | -9.628 | 5 | MAERL066-11 | KC861616 |
|  | CPVP-778 | MM | 01-Jun-10 | COI hap_26 | Ireland (Kerry) | 51.809 | -9.948 | 10 | MAERL121-13 | KC861561 |
|  | CPVP-779 | MM | 01-Jun-10 | COI hap_26 | Ireland (Kerry) | 51.801 | -9.940 | 10 | MAERL052-11 | KC861602 |
|  | CPVP-910 | TW, YF | 10-May-11 | COI hap_26 | France (Brittany) | 48.711 | -3.951 | 11 | MAERL234-13 | KC861530 |
|  | CPVP-901 | TW, YF | 10-May-11 | COI hap_26 | France (Brittany) | 48.711 | -3.951 | 11 | MAERL233-13 | KC861531 |
|  | CPVP-900 | TW, YF | 10-May-11 | COI hap_26 | France (Brittany) | 48.711 | -3.951 | 11 | MAERL182-13 | KC861532 |
|  | CPVP-920 | TW, YF | 13-May-11 | COI hap_26 | France (Brittany) | 47.720 | -4.032 | 15 | MAERL139-13 | KC861543 |
|  | CPVP-921 | TW, YF | 13-May-11 | COI hap_26 | France (Brittany) | 47.720 | -4.032 | 15 | MAERL138-13 | KC861544 |
|  | CPVP-929 | TW, YF | 13-May-11 | COI hap_26 | France (Brittany) | 47.720 | -4.032 | 15 | MAERL137-13 | KC861545 |
|  | CPVP-858 | JG, VP | 10-Mar-11 | COI hap_26 | France (Brittany) | 48.386 | -4.854 | 10 | MAERL129-13 | KC861553 |
|  | CPVP-959 | JG, VP | 13-May-11 | COI hap_26 | France (Brittany) | 48.386 | -4.854 | 10 | MAERL128-13 | KC861554 |
|  | CPVP-961 | JG, VP | 13-May-11 | COI hap_26 | France (Brittany) | 48.386 | -4.854 | 13 | MAERL127-13 | KC861555 |
|  | CPVP-956 | JG, VP | 13-May-11 | COI hap_26 | France (Brittany) | 48.386 | -4.854 | 10 | MAERL038-11 | KC861588 |
|  | CPVP-916 | TW, YF | 13-May-11 | COI hap_26 | France (Brittany) | 47.720 | -4.032 | 15 | MAERL039-11 | KC861589 |
|  | CPVP-912 | TW, YF | 10-May-11 | COI hap_26 / *psb*A hap_1 | France (Brittany) | 48.711 | -3.951 | 11 | **MAERL055-11** | KC861605  (KC819266) |
|  | CPVP-909 | TW, YF | 10-May-11 | COI hap_26 | France (Brittany) | 48.711 | -3.951 | 11 | MAERL056-11 | KC861606 |
|  | CPVP-903 | TW, YF | 10-May-11 | COI hap_26 | France (Brittany) | 48.711 | -3.951 | 11 | MAERL057-11 | KC861607 |
|  | CPVP-906 | TW, YF | 10-May-11 | COI hap_26 | France (Brittany) | 48.711 | -3.951 | 11 | MAERL058-11 | KC861608 |
|  | CPVP-897 | TW, YF | 10-May-11 | COI hap_26 | France (Brittany) | 48.711 | -3.951 | 11 | MAERL059-11 | KC861609 |
|  | CPVP-899 | TW, YF | 10-May-11 | COI hap_26 | France (Brittany) | 48.711 | -3.951 | 11 | MAERL060-11 | KC861610 |
|  | CPVP-1234 | JJ | 22-Jul-11 | COI hap_26 | France (La Rochelle) | 46.233 | -1.381 | 12 | MAERL123-13 | KC861559 |
|  | CPVP-1236 | JJ | 22-Jul-11 | COI hap_26 | France (La Rochelle) | 46.233 | -1.381 | 12 | MAERL122-13 | KC861560 |
|  | **BM000712373**** | WFF | 11-Dec-83 | COI hap_26 | UK (England) | 50.16 | -5.02 |  | MAERL237-13 | KF808323 |
| *Phymatolithon* sp. 1 | CPVP-868 | VP | 11-Mar-11 | COI hap_15 | France (Brittany) | 48.790 | -2.883 | 30 | MAERL172-13 | KC861663 |
|  | CPVP-510 | PN, MR | 23-Feb-11 | COI hap_15 / *psb*A hap_11 | Portugal (Algarve) | 37.046 | -8.336 | 20 | **MAERL069-11** | KC861664  (KC819255) |
| *Phymatolithon* sp. 2 | CPVP-443 | PW | 04-Apr-11 | COI hap_23 / *psb*A hap_7 | Portugal (Madeira) | 32.641 | -16.829 | 18 | **MAERL068-11** | KC861665  (KC819251) |
|  | CPVP-439 | PW | 04-Apr-11 | COI hap_20 | Portugal (Madeira) | 32.641 | -16.829 | 18 | MAERL232-13 | KC861666 |
|  | CPVP-440 | PW | 04-Apr-11 | COI hap_21 | Portugal (Madeira) | 32.641 | -16.829 | 18 | MAERL231-13 | KC861667 |
|  | CPVP-441 | PW | 04-Apr-11 | COI hap_22 / *psb*A hap_7 | Portugal (Madeira) | 32.641 | -16.829 | 18 | **MAERL067-11** | KC861668  (KC819250) |
| *Phymatolithon* sp. 3 | CPVP-627 | IB, RB, VP | 07-Apr-11 | COI hap_1 | Spain (Galicia) | 42.341 | -8.810 | 9 | MAERL168-13 | KC861617 |
|  | CPVP-49 | IB, VP | 19-May-09 | COI hap_1 | Spain (Galicia) | 42.227 | -8.777 | 11 | MAERL078-11 | KC861619 |
|  | CPVP-689 | IB, RB, VP | 05-Apr-11 | COI hap_1 | Spain (Galicia) | 42.258 | -8.751 | 5 | MAERL079-11 | KC861620 |
|  | CPVP-685 | IB, RB, VP | 05-Apr-11 | COI hap_1 | Spain (Galicia) | 42.258 | -8.751 | 5 | MAERL080-11 | KC861621 |
|  | CPVP-686 | IB, RB, VP | 05-Apr-11 | COI hap_1 | Spain (Galicia) | 42.258 | -8.751 | 5 | MAERL081-11 | KC861622 |
|  | CPVP-681 | IB, RB, VP | 05-Apr-11 | COI hap_1 | Spain (Galicia) | 42.258 | -8.751 | 5 | MAERL082-11 | KC861623 |
|  | CPVP-680 | IB, RB, VP | 05-Apr-11 | COI hap_1 | Spain (Galicia) | 42.258 | -8.751 | 5 | MAERL083-11 | KC861624 |
|  | CPVP-695 | IB, RB, VP | 05-Apr-11 | COI hap_1 | Spain (Galicia) | 42.258 | -8.751 | 5 | MAERL084-11 | KC861625 |
|  | CPVP-694 | IB, RB, VP | 05-Apr-11 | COI hap_1 | Spain (Galicia) | 42.258 | -8.751 | 5 | MAERL085-11 | KC861626 |
|  | CPVP-676 | IB, RB, VP | 05-Apr-11 | COI hap_1 / *psb*A hap_9 | Spain (Galicia) | 42.258 | -8.751 | 5 | **MAERL086-11** | KC861627  (KC819260) |
|  | CPVP-622 | IB, RB, VP | 07-Apr-11 | COI hap_1 | Spain (Galicia) | 42.341 | -8.810 | 9 | MAERL170-13 | KC861628 |
|  | CPVP-644 | IB, RB, VP | 05-Apr-11 | COI hap_1 | Spain (Galicia) | 42.212 | -8.896 | 11 | MAERL088-11 | KC861635 |
|  | CPVP-645 | IB, RB, VP | 05-Apr-11 | COI hap_1 | Spain (Galicia) | 42.212 | -8.896 | 11 | MAERL089-11 | KC861636 |
|  | CPVP-646 | IB, RB, VP | 05-Apr-11 | COI hap_1 | Spain (Galicia) | 42.212 | -8.896 | 11 | MAERL090-11 | KC861637 |
|  | CPVP-670 | IB, RB, VP | 05-Apr-11 | COI hap_1 | Spain (Galicia) | 42.212 | -8.896 | 11 | MAERL091-11 | KC861638 |
|  | CPVP-664 | IB, RB, VP | 05-Apr-11 | COI hap_1 | Spain (Galicia) | 42.212 | -8.896 | 11 | MAERL092-11 | KC861639 |
|  | CPVP-669 | IB, RB, VP | 05-Apr-11 | COI hap_1 | Spain (Galicia) | 42.212 | -8.896 | 11 | MAERL093-11 | KC861640 |
|  | CPVP-618 | IB, RB, VP | 07-Apr-11 | COI hap_1 | Spain (Galicia) | 42.341 | -8.810 | 9 | MAERL094-11 | KC861641 |
|  | CPVP-639 | IB, RB, VP | 07-Apr-11 | COI hap_1 / *psb*A hap_9 | Spain (Galicia) | 42.341 | -8.810 | 9 | **MAERL095-11** | KC861642  (KC819258) |
|  | CPVP-1060 | IB, FB, VP | 23-Jun-11 | COI hap_1 | Spain (Galicia) | 42.569 | -8.890 | 4 | MAERL151-13 | KC861644 |
|  | CPVP-1136 | IB, FB, VP | 23-Jun-11 | COI hap_1 | Spain (Galicia) | 42.600 | -8.874 | 3 | MAERL152-13 | KC861645 |
|  | CPVP-1134 | IB, FB, VP | 23-Jun-11 | COI hap_1 | Spain (Galicia) | 42.600 | -8.874 | 3 | MAERL153-13 | KC861646 |
|  | CPVP-611 | IB, RB, VP | 07-Apr-11 | COI hap_1 | Spain (Galicia) | 42.394 | -8.915 | 13 | MAERL154-13 | KC861647 |
|  | CPVP-600 | IB, RB, VP | 07-Apr-11 | COI hap_1 | Spain (Galicia) | 42.394 | -8.915 | 13 | MAERL155-13 | KC861648 |
|  | CPVP-1064 | IB, FB, VP | 23-Jun-11 | COI hap_1 | Spain (Galicia) | 42.569 | -8.890 | 4 | MAERL156-13 | KC861649 |
|  | CPVP-1261 | IB, VGR, VP | 05-Aug-11 | COI hap_8 | Spain (Galicia) | 42.491 | -8.999 | 5 | MAERL157-13 | KC861650 |
|  | CPVP-1260 | IB, VGR, VP | 05-Aug-11 | COI hap_1 | Spain (Galicia) | 42.491 | -8.999 | 5 | MAERL158-13 | KC861651 |
|  | CPVP-1259 | IB, VGR, VP | 05-Aug-11 | COI hap_1 | Spain (Galicia) | 42.491 | -8.999 | 5 | MAERL159-13 | KC861652 |
|  | CPVP-1258 | IB, VGR, VP | 05-Aug-11 | COI hap_8 | Spain (Galicia) | 42.491 | -8.999 | 5 | MAERL160-13 | KC861653 |
|  | CPVP-1257 | IB, VGR, VP | 05-Aug-11 | COI hap_1 | Spain (Galicia) | 42.491 | -8.999 | 5 | MAERL161-13 | KC861654 |
|  | CPVP-1256 | IB, VGR, VP | 05-Aug-11 | COI hap_8 | Spain (Galicia) | 42.491 | -8.999 | 5 | MAERL162-13 | KC861655 |
|  | CPVP-749 | VP, IB, RB | 05-Apr-11 | COI hap_1 | Spain (Galicia) | 42.227 | -8.777 | 11 | MAERL163-13 | KC861656 |
|  | CPVP-748 | VP, IB, RB | 05-Apr-11 | COI hap_1 | Spain (Galicia) | 42.227 | -8.777 | 11 | MAERL164-13 | KC861657 |
|  | CPVP-51 | IB, VP | 19-May-09 | COI hap_1 | Spain (Galicia) | 42.227 | -8.777 | 11 | MAERL165-13 | KC861658 |
|  | CPVP-50 | IB, VP | 19-May-09 | COI hap_1 | Spain (Galicia) | 42.227 | -8.777 | 11 | MAERL166-13 | KC861659 |
|  | CPVP-633 | IB, RB, VP | 07-Apr-11 | COI hap_1 | Spain (Galicia) | 42.341 | -8.810 | 9 | MAERL167-13 | KC861660 |
|  | CPVP-626 | IB, RB, VP | 07-Apr-11 | COI hap_1 | Spain (Galicia) | 42.341 | -8.810 | 9 | MAERL169-13 | KC861662 |
|  | CPVP-501 | PN, MR | 23-Feb-11 | COI hap_13 | Portugal (Algarve) | 37.046 | -8.336 | 20 | MAERL076-11 | KC861661 |
|  | CPVP-452 | PN, MR | 02-Mar-11 | COI hap_10 | Portugal (Algarve) | 37.027 | -8.317 | 20 | MAERL077-11 | KC861618 |
|  | CPVP-480 | PN, MR | 02-Mar-11 | COI hap_12 | Portugal (Algarve) | 37.027 | -8.317 | 20 | MAERL070-11 | KC861629 |
|  | CPVP-478 | PN, MR | 02-Mar-11 | COI hap_12 / *psb*A hap_9 | Portugal (Algarve) | 37.027 | -8.317 | 20 | **MAERL071-11** | KC861630  (KC819253) |
|  | CPVP-451 | PN, MR | 02-Mar-11 | COI hap_1 | Portugal (Algarve) | 37.027 | -8.317 | 20 | MAERL072-11 | KC861631 |
|  | CPVP-453 | PN, MR | 02-Mar-11 | COI hap_1 | Portugal (Algarve) | 37.027 | -8.317 | 20 | MAERL073-11 | KC861632 |
|  | CPVP-503 | PN, MR | 23-Feb-11 | COI hap_13 | Portugal (Algarve) | 37.046 | -8.336 | 20 | MAERL074-11 | KC861633 |
|  | CPVP-479 | PN, MR | 02-Mar-11 | COI hap_13 | Portugal (Algarve) | 37.027 | -8.317 | 20 | MAERL075-11 | KC861634 |
|  | CPVP-77 | IB, VP, EB, PN, RS | 04-Sep-08 | COI hap_9 | Portugal (Algarve) | 37.110 | -8.640 | 15 | MAERL150-13 | KC861643 |
| *Phymatolithon* sp. 4 | CPVP-502 | PN, MR | 23-Feb-11 | COI hap_14 / *psb*A hap_10 | Portugal (Algarve) | 37.046 | -8.336 | 20 | **MAERL087-11** | KC861669  (KC819254) |

^1^* = collections from type/neotype localities; ** = holotype/neotype specimen.

^2^Abbreviations for collectors include: ARE, Alfonso Ramos-Esplá; CP, Cristina Pardo; CS, Carlos Sangil; DP, Daniel Pritchard; EB, Estíbaliz Berecíbar; FB, Francis Bunker; HH, Halldor Halldorsson; IB, Ignacio Bárbara; JB, Jan Buedenbender; JG, Jacques Grall; JH, Jazmin Hernández; JHS, Jason M. Hall-Spencer; JJ, Jerome Jourde; JR, Jan Rueness; KC, Ken Collins; LK, Louise Kregting; MM, Meadhbh Moriarty; MR, Miguel Rodrigues; NK, Nick Kamenos; NN, Niamh Nolan; PN, Pedro Neves; PW, Peter Wirtz; RB, Rodolfo Barreiro; RS, Rui Santos; TW, Thomas Wilfried; VGR, Verónica García Redondo; VH, Vivian Husa; VP, Viviana Peña; WFF, W. F. Farnham; YF, Yann Fontana.

^3^BOLD sequence page ID. Detailed collection data can be acquired from BOLDSYSTEMS ([www.boldsystems.org](http://www.boldsystems.org)).

^4^IDs in bold and underlined indicate vouchers sequenced for both COI-5P and *psb*A markers.

^5^Accession number for *psb*A fragments in parenthesis; otherwise, accession numbers refer to COI-5P sequences.
