# Supplementary material for: A Multilocus Species Delimitation Reveals a Striking Number of Species of Coralline Algae Forming Maerl in the OSPAR Maritime Area
Source: PLoS One. 2014 Aug 11;9(8):e104073. doi: 10.1371/journal.pone.0104073 (PMC4128821; doi:10.1371/journal.pone.0104073)
Supplement: Table S2 — A. COI-5P sequence divergence within (diagonal) and between (below diagonal) the PSHs shown in Fig. 1. B. COI-5P sequence divergence within (diagonal) and between (below diagonal) the SSHs shown in Fig. 2. (PDF) [file pone.0104073.s002.pdf]

Table S2A. COI-5P sequence divergence within (diagonal) and between (below diagonal) the PSHs shown in Fig. 1. The number of base substitutions per site from averaging over all sequence pairs between PSHs are shown. Analyses were conducted using the Kimura 2-parameter model [1]. In bold, values below an arbitrary 2% cutoff. n/c = not computable.

	PSH1	PSH2	PSH3	PSH4	PSH5	PSH6	PSH7	PSH8	PSH9	PSH10	PSH11	PSH12	PSH13	PSH14	PSH15	PSH16
**PSH1**	**0.002**															
**PSH2**	0.144	n/c														
**PSH3**	0.141	0.041	n/c													
**PSH4**	0.168	0.169	0.167	n/c												
**PSH5**	0.168	0.171	0.169	**0.011**	n/c											
**PSH6**	0.167	0.139	0.139	0.057	0.064	n/c										
**PSH7**	0.166	0.141	0.141	0.056	0.063	**0.002**	**0.002**									
**PSH8**	0.173	0.154	0.156	0.114	0.117	0.123	0.122	**0.002**								
**PSH9**	0.168	0.153	0.155	0.109	0.113	0.118	0.118	**0.005**	n/c							
**PSH10**	0.185	0.154	0.145	0.158	0.167	0.145	0.144	0.157	0.156	n/c						
**PSH11**	0.179	0.141	0.141	0.147	0.156	0.138	0.141	0.145	0.145	0.028	**0.003**					
**PSH12**	0.192	0.159	0.159	0.146	0.154	0.143	0.142	0.154	0.149	0.081	0.068	**0.006**				
**PSH13**	0.156	0.160	0.153	0.151	0.157	0.142	0.141	0.165	0.158	0.135	0.133	0.137	**0.004**			
**PSH14**	0.165	0.169	0.157	0.148	0.154	0.146	0.145	0.162	0.155	0.143	0.141	0.145	0.022	n/c		
**PSH15**	0.198	0.199	0.193	0.192	0.190	0.186	0.185	0.189	0.188	0.186	0.174	0.188	0.189	0.182	n/c	
**PSH16**	0.180	0.193	0.184	0.188	0.184	0.184	0.183	0.179	0.178	0.182	0.167	0.186	0.185	0.173	0.036	n/c

Table S2B. COI-5P sequence divergence within (diagonal) and between (below diagonal) the SSHs shown in Fig. 2. The number of base substitutions per site from averaging over all sequence pairs between PSHs are shown. Analyses were conducted using the Kimura 2-parameter model [1]. In bold, values below an arbitrary 2% cutoff. n/c = not computable.

	**SSH1**	**SSH2**	**SSH3**	**SSH4+5**	**SSH6+7**	**SSH8+9**	**SSH10**	**SSH11**	**SSH12**	**SSH13**	**SSH14**	**SSH15**	**SSH16**
**SSH1**	**0.002**												
**SSH2**	0.144	n/c											
**SSH3**	0.141	0.041	n/c										
**SSH4+5**	0.168	0.170	0.168	**0.011**									
**SSH6+7**	0.167	0.141	0.141	0.060	**0.002**								
**SSH8+9**	0.171	0.154	0.156	0.114	0.121	**0.004**							
**SSH10**	0.185	0.154	0.145	0.162	0.144	0.157	n/c						
**SSH11**	0.179	0.141	0.141	0.152	0.140	0.145	0.028	**0.003**					
**SSH12**	0.192	0.159	0.159	0.150	0.142	0.152	0.081	0.068	**0.006**				
**SSH13**	0.156	0.160	0.153	0.154	0.141	0.163	0.135	0.133	0.137	**0.004**			
**SSH14**	0.165	0.169	0.157	0.151	0.145	0.160	0.143	0.141	0.145	0.022	n/c		
**SSH15**	0.198	0.199	0.193	0.191	0.185	0.188	0.186	0.174	0.188	0.189	0.182	n/c	
**SSH16**	0.180	0.193	0.184	0.186	0.183	0.179	0.182	0.167	0.186	0.185	0.173	0.036	n/c

[1] Kimura M (1980) A simple method for estimating evolutionary rates of base substitutions through comparative studies of nucleotide-sequences. J Mol Evol 16: 111-120.
