# Supplementary material for: A Multilocus Species Delimitation Reveals a Striking Number of Species of Coralline Algae Forming Maerl in the OSPAR Maritime Area
Source: PLoS One. 2014 Aug 11;9(8):e104073. doi: 10.1371/journal.pone.0104073 (PMC4128821; doi:10.1371/journal.pone.0104073)
Supplement: Table S3 — Matches with our sequences in public databases (Genbank: COI-5P and psbA; BOLD: COI-5P). (PDF) [file pone.0104073.s003.pdf]

Table S3. Matches with our sequences in public databases (Genbank: COI-5P and *psb*A; BOLD: COI-5P). Only hits with an identity beyond a minimum threshold are reported for Genbank (98% for COI-5P, 99% for psbA) while BOLD's identification engine determines those queries that provide an acceptable match. Query coverage (i.e. overlap) always was >92%. BOLD searches were conducted against all barcode records lodged on BOLD in February 12th, 2013 (1,395,901 sequences); this unvalidated library includes records without species level identification, species represented by only one or two specimens, and species with interim taxonomy. Barcode Index Numbers (BIN) are clusters of similar sequences uploaded to BOLD that are likely to correspond to biological species.

Query	Genbank best hits					BOLD best hits			
	Accession no.	Identity	Taxonomy	Voucher	Location	BIN no. (no. collections)	Identity	Taxonomy	Location
					COI-5P				
SSH4+5 (Hap7)	HM918812	99.2%	*Lithothamnion glaciale*	GWS007542	Canada (Newfoundland and Labrador, English Harbour Eastern Cove) (47.633N 54.87W)	BOLD:AAA6958 (39 collect.)	98.9-99.9%	*Lithothamnion glaciale*	USA (Massachusetts, Maine); Canada (New Brunswick, Manitoba, Newfoundland and Labrador, Nova Scotia)
SSH4+5 (Hap24)	HM918812	98.8%	*Lithothamnion glaciale*	GWS007542	Canada (Newfoundland and Labrador, English Harbour Eastern Cove) (47.633N 54.87W)	BOLD:AAA6958 (39 collect.)	98.6-99.2%	*Lithothamnion glaciale*	USA (Massachusetts, Maine); Canada (New Brunswick, Manitoba, Newfoundland and Labrador, Nova Scotia)
SSH6+7 (Hap5)	No match					BOLD:ABA9580 (2 collect.)	99.9%	Corallinac sp.29BCcrust	Canada (Bristish Columbia)
SSH6+7 (Hap17)	No match					BOLD:ABA9580 (2 collect.)	100%	Corallinac sp.29BCcrust	Canada (Bristish Columbia)
SSH6+7 (Hap19)	No match					BOLD:ABA9580 (2 collect.)	99.9%	Corallinac sp.29BCcrust	Canada (Bristish Columbia)

Query	Genbank best hits					BOLD best hits			
	Accession no.	Identity	Taxonomy	Voucher	Location	BIN no. (no. collections)	Identity	Taxonomy	Location
SSH12 (Hap26)	GQ917247	100%	Uncultured Corallinales	LBC0001[a]	France (47.63905N 3.4166667W)	No match			
SSH12 (Hap28)	GQ917247	99.8%	Uncultured Corallinales	LBC0001[a]	France (47.63905N 3.4166667W)	No match			
SSH12 (Hap29)	GQ917247	99.2%	Uncultured Corallinales	LBC0001[a]	France (47.63905N 3.4166667W)	No match			
SSH16 (Hap18)	GQ917510	99.9%	Uncultured Corallinales	LBC004	France (47.63905N 3.4166667W)	No match			
					***psb*A**				
SSH4+5 (91)	JQ422235	99.8%	*Lithothamnion glaciale*	GWS007542	N/A	N/A			
SSH4+5 (1444)	JQ422235	99.7%	*Lithothamnion glaciale*	GWS007542	N/A	N/A			
SSH 12 (47)	GQ917708	100%	Uncultured Corallinales	LBC0013	France (48.731937N 3.939152W)	N/A			

[a] Voucher LBC0001 has been subsequently assigned to *Phymatolithon* sp. by Bittner *et al*. (2011)
